# A mixture of carcinoid tumors, extensive neuroendocrine proliferation, and multiple pulmonary sclerosing hemangiomas

**DOI:** 10.1186/1477-7819-12-209

**Published:** 2014-07-15

**Authors:** Yihong Wang, Qicai He, Wei Shi, Jun Wang, Hongxiu Ji

**Affiliations:** 1 Department of Surgery and Pathology, Sir Run Run Shaw Hospital, Zhejiang University College of Medicine, 3# East Qingchun Road, Hangzhou, Zhejiang 310016, China; 2 Department of Thoracic Surgery, Sir Run Run Shaw Hospital, Zhejiang University College of Medicine, 3# East Qingchun Road, Hangzhou, Zhejiang 310016, China; 3 Department of Pathology and Laboratory Medicine, Loma Linda University Medical Center, 11234 Anderson Street Loma, Linda, CA 92354, USA; 4 Department of Pathology, Overlake Hospital Medical Center, Bellevue, WA 98004, USA

**Keywords:** Pulmonary sclerosing hemangioma, Multiple, Carcinoid, Pulmonary neuroendocrine cell hyperplasia

## Abstract

We encountered an extremely rare case of multiple pulmonary sclerosing hemangiomas (PSH) with extensive neuroendocrine lesions, including pulmonary neuroendocrine cell (PNC) hyperplasia, multiple carcinoid tumorlets and typical carcinoid tumors within one pulmonary lobe. To the best of our knowledge, this is the first reported case in the English medical literature of PSH combined and admixed with carcinoid tumors and extensive neuroendocrine proliferation. This case is noteworthy for several reasons. First, the lesion is multi-nodular and unusually large for a typical PSH, which may mimic malignancy on imaging studies and cause diagnostic difficulties. Second, sampling bias may lead to diagnostic errors for a lesion containing two different types of neoplasms. Third, our case displays a rare mixed and mosaic pattern of PSH with a full spectrum of pulmonary neuroendocrine lesions, which may imply a potential intrinsic association in pathogenesis between PSH and concomitant neuroendocrine neoplasms. The clinical implication of multiple PSHs is also discussed.

## Background

Pulmonary sclerosing hemangioma (PSH) was first described by Liebow and Hubbell in 1956 as an uncommon neoplasm originally hypothesized of to be vascular origin [1]. It typically presents as a solitary and slow-growing mass, demonstrating a benign clinical course. However, multiplicity could be a very unusual presentation and its histopathogenesis and biological behavior are still unclear [2-5]. PSH consists of two distinctive types of tumor cells, the surface cuboidal cells and the round stromal cells, forming a mixture of sclerotic, hemorrhagic, papillary and solid histologic patterns. Given the unique constellation of two types of tumor cells respectively harboring different histological features of PSH, a variety of pathogenesis theories other than vascular origin was postulated, including mesothelial, mesenchymal and epithelial origin. Some previous studies revealed neuroendocrine features in certain PSHs, suggesting the possibility of their neuroendocrine origin [6,7]. To date, most scholars have reached the consensus that PSH derives from primitive respiratory epithelium [8] while the mechanism behind its neuroendocrine differentiation remains controversial. Although rare, carcinoid tumorlets or typical carcinoid tumors have been previously described as concomitant with PSH, but as separate lesions [8,9]. Whether this incidental concurrence shares a common underlying cause is still a mystery. A PSH intimately mixed with multiple carcinoid tumorlets or typical carcinoid tumors has never been reported in the English medical literature. Here we describe a case of PSH with a full spectrum of pulmonary neuroendocrine lesions, which includes pulmonary neuroendocrine cell hyperplasia, multiple carcinoid tumorlets and carcinoid tumors.

## Case presentation

**Figure 1:**
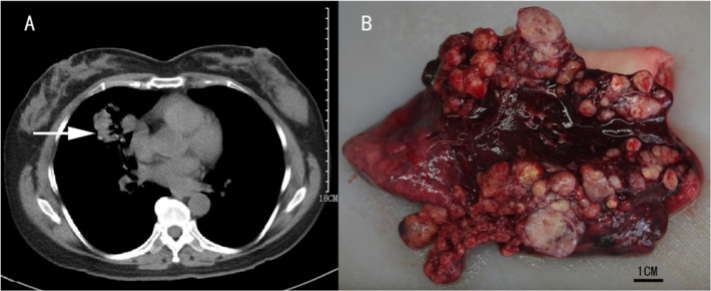
**A solid, gray-to-white legional area in right middle lobe lung. (A)** Computed tomography scan demonstrates nodular and irregular density (arrow) in the medial segment of right middle lobe, with relatively smooth borders and focal dot-like calcifications. **(B)** Resected lung specimen shows multiple nodules of varying sizes seen both beneath subpleural and within the pulmonary parenchyma. The overall lesion measures 9.0 cm in greatest dimension and is composed of nodules ranging from 0.3 cm to 2.2 cm.

The patient was a 50-year old non-smoking Chinese woman who presented with recurrent dry cough with chest pain and dyspnea for over 7 years. Her symptoms aggravated in the recent two weeks. She had never lived in high altitude areas and had no history of lung diseases. Physical examination and laboratory results were unremarkable. A pre-operative chest computed tomography (CT) revealed multiple sheet-like densities in the right middle lobe, with relatively smooth borders and calcified dots in focal areas (Figure 1A). The multiple nodular fashion of the lesion aroused suspicion for malignancy. A subsequent CT-guided fine needle biopsy showed pulmonary neuroendocrine cell (PNC) hyperplasia. This was followed by an open thoracotomy with right middle lobectomy, which was notable for a large multi-nodular lesion measuring 12 cm, occupying the subpleura and pulmonary parenchyma (Figure 1B). No gross pleural, hilar or mediastinal dissemination was detected during surgery.

**Figure 2:**
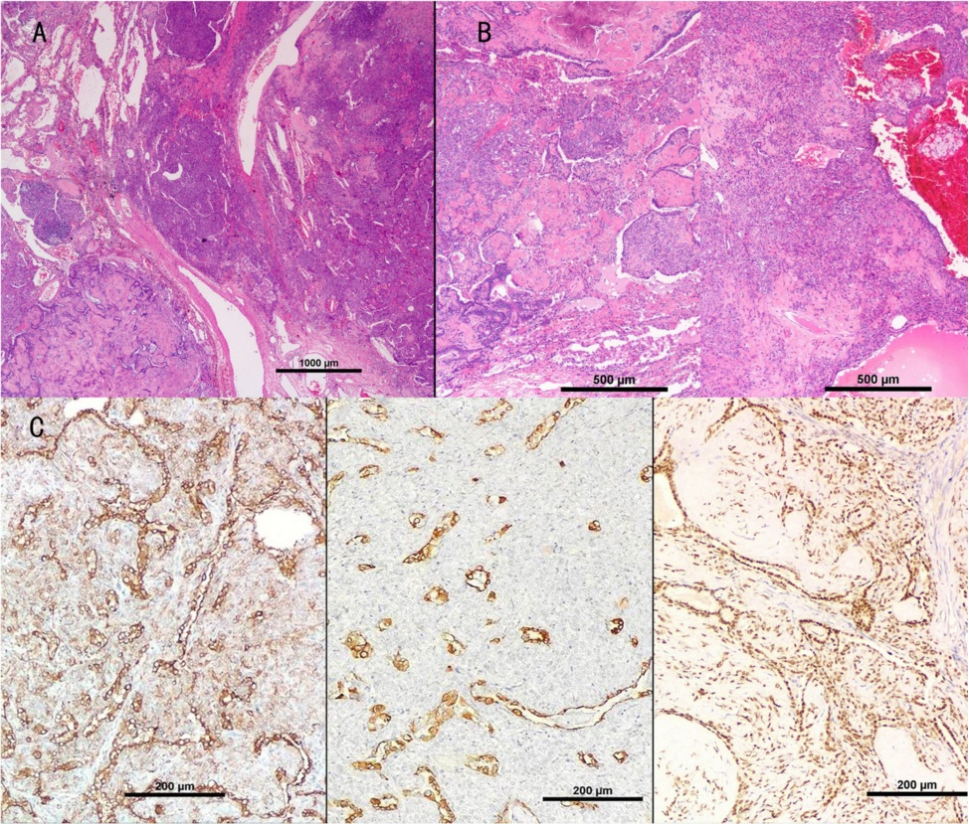
**Histologic and immunohistochemistry findings of pulmonary sclerosing hemangioma (PSH). (A)** Multi-nodular PSH, with fibrous septae dividing different nodules. **(B)** Various histologic patterns of PSH (papillary, sclerotic, solid and hemorrhagic) (H&E stain, original magnification, **(A)** × 20, **(B)** × 40). **(C)** Characteristic immunostaining profile of PSH, both surface cells and round stromal cells are positive for epithelial marker epithelial membrane antigen (left panel) and thyroid transcription factor-1 (right panel), but only surface cells are positive for pancytokeratin (another epithelial marker) (middle panel) (Polymer method; original magnification, ×100).

**Figure 3:**
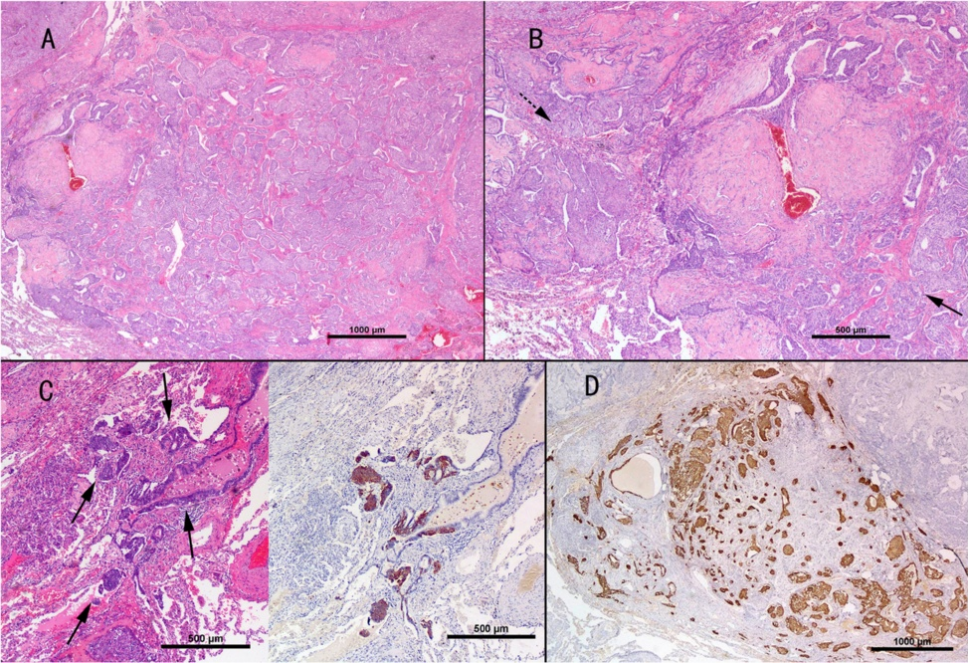
**Spectrum of pulmonary neuroendocrine cell (PNC) proliferation and its intimate association with PSH. (A)** Typical carcinoid tumor, 5.5 mm in size, composed of uniform short-spindled or oval shaped cells. **(B)** Carcinoid tumorlet admixed with PSH in a single nodule, with PSH on the left (dotted arrow), tumorlet on the right (arrow). **(C)** Foci of PNC proliferation highlighted by synaptophysin staining beneath airway epithelium (arrows). **(D)** Positive chromogranin staining in a tumorlet, in contrast to surrounding negative PSH areas (H&E stain and polymer method, original magnification, **(A)** and **(D)** × 20,** (B)** and **(C)** × 40).

Macroscopically, the right middle lobe of lung measured 12.2 × 6 × 3.8 cm, with a solid, gray-to-white regional area of approximately 9.0 × 4.2 × 2.0 cm. There were multiple rubbery, hard, round nodules of varying sizes seen both beneath the pleura and within the pulmonary parenchyma. The nodules were well-circumscribed and unencapsulated, measuring from 0.3 cm to 2.2 cm and some of these merged into each other. The cut surface was gray-white, solid, with partially hard areas in the larger nodules.Microscopically, the neoplasm consisted of multiple variable-sized nodules (Figure 2A), some of which coalesced. Most of the lesion displayed a typical architecture of PSH, which generally comprises four histologic patterns: sclerotic, hemorrhagic, papillary and solid (Figure 2B). In the sclerotic areas, scattered calcifications were identified resulting from calcium deposition in sclerotic fibrous stroma. A constellation of two types of tumor cells were found in these areas. One type was the surface cuboidal cells lining papillary or glandular structures and the other was the relatively uniform round to polygonal cells beneath the epithelial cells or arranged as solid sheets or nests. There were abundant and delicate capillaries directly connected to the round neoplastic cells with very scanty fibrous stroma. The cuboidal cells were positive for pancytokeratin, thyroid transcription factor-1 (TTF-1) and epithelial membrane antigen (EMA), whereas the round cells were pancytokeratin negative, but TTF-1 and EMA positive by immunohistochemistry (Figure 2C).Also noted scattered within and intermingled with the PSH, was a distinct spectrum of diffuse PNC proliferation. This included lesions of typical carcinoid tumors (>5 mm) (Figure 3A), carcinoid tumorlets (lesions 2 to 5 mm) and extensive neuroendocrine cell hyperplasia (Figure 3B,C). Extensive minute foci of neuroendocrine cell proliferation were found within or closely adjacent to the PSH structure. No PNC proliferation was appreciated in the pulmonary lobe without presence of PSH.The cells in the neuroendocrine lesions showed immunohistochemical reactivity with TTF-1, chromogranin A (CgA), synaptophysin (SyN) and CD56, but were negative for pancytokeratin or S-100. Some hyperplasic foci of neuroendocrine cells were situated beneath but confined to the airway epithelium, while others penetrated through the basement membrane and infiltrated the lung parenchyma to form multiple nodules and cellular nests with variable sizes, some of which display rosette features. Most PNC neoplasms measured from 2.0 to 4.0 mm, with two of them measured 5.2 mm and 5.5 mm, respectively, which qualified for carcinoid tumors. No mitosis or necrosis was found in the carcinoid tumors. A Ki-67 index was low (<2%,) excluding higher grade neuroendocrine carcinoma, such as atypical carcinoid tumor and small cell carcinoma. In contrast, the surface epithelial and round stromal cells of PSH area were completely negative for neuroendocrine markers, which are CgA, SyN and CD56 (Figure 3D). There was no metastasis found in hilar and mediastinal lymph nodes. The patient received no adjuvant therapy after surgery and has remained disease-free during a 14-month follow-up.

## Discussion

This is a rare case of multiple neuroendocrine tumors and extensive neuroendocrine cell proliferation, not only concurrent with but also located within PSH. PSH was first described by Liebow and Hubbell in 1956 as an uncommon neoplasm of vascular origin [1] and different scholars have since investigated its nature in terms of clinical presentation, morphology, phenotype and prognosis thereafter. Its clinical symptoms include cough, hemoptysis, chest pain and dyspnea although most patients are asymptomatic, their tumors being identified incidentally. It typically affects middle-aged adults with female predominance, presents as a solitary and slow growing mass located in the lung periphery, demonstrating a benign clinical course [8]. However, occasional recurrence and progressive pleural dissemination in post-operative follow-up of PSH patients have been previously reported [10,11]. Regional lymph node metastasis and rare locally infiltrative growth to pleura may suggest low malignant potential for the minority of PSH [10-13]. Histologically, PSH consists of two types of tumor cells, the surface cuboidal cells and the round stromal cells, forming a mixture of sclerotic, hemorrhagic, papillary and solid patterns. By immunohistochemistry, both the surface cell and round cell populations express TTF-1 and EMA in the majority of cases, with only surface cells exhibiting pancytokeratin positivity. As suggested by a large series of immunohistochemistry analyses [6], most scholars have reached the consensus that PSH derives from primitive respiratory epithelium. In addition, some recent molecular studies further confirmed the clonal and neoplastic nature of both components of PSH and their common origin [14-17].

Given the mixed appearance of PSH and PNC lesions in our case, it was first assumed that it may represent a ‘collision tumor’, that is a PSH coincidentally developing in a lung lobe that contains diffuse PNC hyperplasia, such as in the very rare condition of diffuse idiopathic neuroendocrine cell hyperplasia (DIPNECH), a preinvasive precursor to tumorlets and carcinoid tumors and defined by the World Health Organization. DIPNECH develops without predisposing conditions and affects similar populations as PSH, which are non-smoking and middle-aged women [8,18-20]. However, PNC hyperplasia is also observed in individuals who live at high altitude, in cigarette smokers, in various chronic lung diseases and associated with distortion of the lung microstructure and with hypoxia, but the definite cause for hyperplasia is just beginning to be understood [18,21]. Thus, another possible explanation for ‘collision’ is that PSH tumor cells, devoid of normal respiratory epithelium function, somehow disrupted local airway homeostasis and caused damage to adjacent alveolar epithelium. The slow-progressing course of the neoplasm delivered prolonged insult to the airway epithelium in the similar fashion as other forms of chronic lung diseases. PNCs, as providers of the airway epithelium stem cell niche [22,23], may be activated to proliferate in order to restore the damaged epithelium. In addition, decreased pulmonary compliance and ventilation disorder could occur as PSH grows to a considerable size and lead to local intrapulmonary hypoxia. It is possible that PNCs, known as chemoreceptors sensitive to hypoxia [24] would then be stimulated to overproduce various bioactive peptides, including gastrin-releasing peptide, which may in return promote further proliferation and differentiation of PNCs [21,22]. Moreover, hypoxia may trigger a cascade of molecular events that can drive tumorigenesis of pulmonary carcinoids [25,26]. Over time, PSH could induce excessive PNC proliferation, which would then manifest clinically as mixed PSH and PNC tumors.

However, the complex morphologic finding in our case may also suggest that it may represent ‘one’ tumor, an example of clonal expansion of a primitive respiratory epithelial cell that is capable of divergent cellular differentiation, not only to the two distinct cell types of PSH, namely, surface cuboidal cells and round stromal cells, but also to neuroendocrine cells as the ‘third’ cell type. In certain circumstances, the same molecular event of PSH could facilitate the neuroendocrine phenotype differentiation of respiratory epithelial progenitors of multipotency. The hypothesis is made based upon following observations in our case: 1) although morphologically distinctive, the two different components are so well mingled that they cannot be distinguished either by radiology or gross examination; 2) intimate mixture of PSH and extensive PNC lesions are present throughout the entire multi-nodular lesion; 3) the two different components both express TTF-1 (a tissue-specific transcription factor expressed by type II pneumocytes and Clara cells). This ‘one tumor’ theory was further suggested by previous studies demonstrating different phenotype and differentiation status of the two types of PSH cells and neuroendocrine immunoactivity and neurosecretory granules in nested cells of PSH [6-8,17]. In addition, the recent finding that PNCs share a common lineage with alveolar cells [22] may further support that possibility that this rare ‘third’ cell type and the other two may originate from the same progenitor. This ‘one tumor’ theory may be further validated if identical molecular events could be shown in both PSH and PNC components. Interestingly, the one and only reported case of carcinoid tumors associated with PSH in the literature described carcinoid tumors as completely separate from PSH, with absence of PNC hyperplasia or carcinoid tumorlets and completely negative staining for TTF-1 in carcinoid tumor cells [9], which is different from our case and may indicate other pathogenetic pathways.

Another intriguing feature of our case is the multiple nodular presentation and unusually large size of PSH, which could cause diagnostic difficulties for both clinicians and radiologists. The sampling bias caused by the large lesional area and multi-nodular growth pattern might lead to pre-operative diagnostic errors for a lesion containing more than one tumor component. In our case, the CT-guided core needle biopsy only showed foci of PNC hyperplasia without a component of diagnostic PSH and no confirmative diagnosis was made until after surgical resection. Multiple PSH is rare, accounting only for 3 to 4% of all PSHs in large series and case reviews [3,5,11,27]. The multiple tumors could locate within one or more than one pulmonary lobe, unilaterally or bilaterally or even in pulmonary fissures or the mediastinum, mimicking malignancies or metastatic diseases. [3,5,27,28]. Those tumors were usually completely resected by lobectomy or pulmonary wedge resection. So far, there were no reported cases of lymph node metastasis, distal spread or malignant transformation. Whether they were multicentric in origin or intrapulmonary spread from one primary lesion is still unclear. Multiple PSHs have also been reported to be associated with atypical adenomatous hyperplasia, which is considered to be a forerunner of pulmonary adenocarcinoma [3,27]. Accumulation of such cases is needed to further elucidate the histogenesis and biological behavior of this rare entity.

## Conclusion

To our knowledge, this is the first described case of mixed carcinoid and extensive neuroendocrine proliferation with multiple PSHs. Our patient received no post-operative chemotherapy or radiation. No recurrence or metastasis was identified during a 14-month follow-up. Although PSH is generally considered a benign tumor, the multi-nodular growth pattern and concurrence of extensive PNC lesions in this case would justify long-term post-operative surveillance. Whether PNC neoplasms and PNC hyperplasia present in PSH as a simple combination or a true component still remains a mystery. Further understanding of the molecular pathogenesis of these diseases and more studies of similar tumor-combining cases are required to unveil their potential intrinsic correlation or cross-entity interaction. Although rare, PSH could present in a multiple nodular form, mimicking malignancy in imaging studies. Due to its rarity, the clinical behavior of multiple PSHs is not yet well understood and further studies are needed to decide whether the lesions are multicentric in origin or a dissemination/metastasis from one primary lesion.

## Consent

Written informed consent was obtained from the patient for publication of this Case Report and any accompanying images. A copy of the written informed consent is available for review by the Editor-in-Chief of this journal.

## Abbreviations

PSH: pulmonary sclerosing hemangiomas; PNC: pulmonary neuroendocrine cell; TTF-1: thyroid transcription factor-1; EMA: epithelial membrane antigen; CgA: chromogranin A; SyN: synaptophysin; DIPNECH: diffuse idiopathic neuroendocrine cell hyperplasia.

## Competing interests

The authors declare that they have no competing interests.

## Authors' contributions

YW participated in the design of the study and drafted the manuscript. QH made contributions to the acquisition and analysis of clinical data. SW made contributions towards analyzing the histological features of this case by H&E and immunohistochemistry staining. JW and HJ revised the manuscript critically for important intellectual content. All authors read and approved the final manuscript.
